# Parboiled Germinated Brown Rice Improves Cardiac Structure and Gene Expression in Hypertensive Rats

**DOI:** 10.3390/foods12010009

**Published:** 2022-12-20

**Authors:** Nattira On-Nom, Kanoknad Khaengamkham, Aikkarach Kettawan, Thanaporn Rungruang, Uthaiwan Suttisansanee, Piya Temviriyanukul, Pattaneeya Prangthip, Chaowanee Chupeerach

**Affiliations:** 1 Institute of Nutrition, Mahidol University, Salaya, Phuttamonthon, Nakhon Pathom 73170, Thailand; 2 Department of Anatomy, Faculty of Medicine Siriraj Hospital, Mahidol University, Bangkok 10700, Thailand; 3 Department of Tropical Nutrition and Food Science, Faculty of Tropical Medicine, Mahidol University, Ratchathewi, Bangkok 10400, Thailand

**Keywords:** Khao Dawk Mali 105, blood pressure, fibrosis, Sprague-Dawley rat, renin angiotensin system

## Abstract

Hypertension leads to oxidative stress, inflammation, and fibrosis. The suppression of these indicators may be one treatment approach. Parboiled germinated brown rice (PGBR), obtained by steaming germinated Jasmine rice, reduces oxidative stress and inflammation in vivo. PGBR contains more bioactive compounds than brown rice (BR) and white rice (WR). Anti-hypertensive benefits of PGBR have been predicted, but research is lacking. The anti-hypertensive effects of PGBR were investigated in the downstream gene network of hypertension pathogenesis, including the renin–angiotensin system, fibrosis, oxidative stress production, and antioxidant enzymes in *N*-nitro-L-arginine methyl ester (L-NAME)-induced hypertensive rats. To strengthen our findings, the cardiac structure was also studied. PGBR-exposed rats showed significant reductions in systolic blood pressure (SBP) compared to the hypertensive group. WR did not reduce SBP because of the loss of bioactive compounds during intensive milling. PGBR also reduced the expression of the angiotensin type 1 receptor (AT1R), transforming growth factor-β (TGF-β), and nicotinamide adenine dinucleotide phosphate (NADPH) oxidase (NOX4), which contribute to the renin–angiotensin system, fibrosis, and oxidative stress production, respectively. Losartan (Los, an anti-hypertensive drug)-treated rats also exhibited similar gene expression, implying that PGBR may reduce hypertension using the same downstream target as Los. Our data also indicated that PGBR reduced cardiac lesions, such as the cardiomyopathy induced by L-NAME. This is the first report on the anti-hypertensive effects of PGBR in vivo by the suppression of the renin response, fibrosis, and improved cardiac structure.

## 1. Introduction

Hypertension, or high blood pressure, is a serious medical condition that leads to vascular disease, which is the leading cause of death worldwide [1]. The molecular process by which hypertension impacts health is complicated. Simply stated, the sympathetic nervous system (SNS) induces the release of renin, also known as angiotensinogenase, from the kidney, which then hydrolyzes angiotensinogen to produce angiotensin I (Ang I), which is then hydrolyzed by lung angiotensin-converting enzymes (ACEs), yielding angiotensin II (Ang II), a key factor in hypertension and myocardial remodeling. Ang II is a major bioactive peptide contributing to the renin–angiotensin system (RAS) and increases blood pressure by activating the angiotensin II type 1 receptor (AT1R), leading to vascular smooth muscle contraction [2]. Ang II has also been reported to stimulate transforming growth factor-β (TGF-β) into a potent stimulator of collagen-producing cardiac fibroblasts [3,4]. Hypertension provokes oxidative stress by activating a major ROS-producing enzyme, nicotinamide adenine dinucleotide phosphate (NADPH) oxidase, which then activates the redox-sensitive transcription factor nuclear factor kappa B (NF-κB). The activated NF-κB is then translocated to the nucleus and promotes the transcription of several inflammation-associated genes [5]. Finally, prolonged hypertension with tissue damage may result in the accumulation of collagen type I (Col I) and collagen type III (Col III), which are biomarkers of changed cardiac structure and fibrosis [6]. Thus, hypertension has extensive impacts on gene networks involving inflammation, antioxidant enzymes, and fibrosis. The low-grade inflammation resulting from hypertension can chronically damage cells and organs within the body, leading to other health complications such as stroke, renal failure, and dementia [7].

Jasmine rice is Thailand’s primary export rice and is renowned for its distinctive flavor and texture. Jasmine rice is typically consumed as white rice; however, white Jasmine rice has a low nutritive value and bioactive compounds with a high glycemic index due to the intensive milling, which removes the rice husk, germ, and bran. By contrast, brown Jasmine rice with the rice germ has a high nutritive value and bioactive compounds with a low glycemic index [8]. Parboiling germinated brown rice increases the bioactive compounds, especially γ-aminobutyric acid (GABA) and total phenolics [9]. Previous studies demonstrated that parboiled germinated brown rice (PGBR), produced from Khao Dawk Mali 105 (*Oryza sativa* L. ssp. *indica* cv. KDML 105) had a higher nutritive value and more bioactive components such as GABA, ferulic acid, γ-oryzanol, γ-tocotrienol, and fiber than brown rice (BR) and white rice (WR) [10]. PGBR also prevented carbon tetrachloride (CCl_4_)-induced liver oxidative stress and injury by enhancing the antioxidant capacities in rats [9]. The consumption of PGBR made from KDML 105 also reduced liver inflammation and fibrosis in vivo [11].

The modulation of oxidative stress, inflammation, and fibrosis might be a therapeutic target for hypertension, but the efficacy of PGBR as an anti-hypertensive agent that reduces oxidative stress and inflammation remains questionable. Therefore, this study explored the anti-hypertensive effects of PGBR on a network of genes involved in hypertension complications and cardiac structure. The genes were divided into four groups, including (i) the renin–angiotensin system, such as AT1R; (ii) the TGF-β, collagen type I (Col I), and collagen type III (Col III) genes, which are involved in fibrosis; (iii) the NADPH oxidase (NOX4) gene, which is involved in oxidative stress production; and (iv) antioxidant enzymes and nitric oxide-producing enzymes (glutathione peroxidase (GPx), catalase (CAT), superoxide dismutase (SOD), and endothelial nitric oxide synthase (eNOS)). To strengthen the evidence of PGBR as a promising hypertensive agent, we also investigated the cardiac structure in hypertensive rats. Our findings can be used to enhance the development of functional foods derived from PBGR as a strategy for controlling hypertension.

## 2. Materials and Methods

### 2.1. Animals and Diet

Male Sprague-Dawley rats (200–250 g) were obtained from the National Laboratory Animal Center, Mahidol University, Thailand. The rats were maintained in an environmentally controlled room (23 ± 2 °C, with a 12 h light/12 h dark cycle) and given access to food and water ad libitum. All experiments were conducted according to protocols approved by the Siriraj Animal Care and Use Committee (SiACUC) from the Faculty of Medicine of Siriraj Hospital, Mahidol University, and complied with international guidelines for animal research protection, such as the International Guiding Principles for Biochemical Research Involving Animals (SiACUP number: SI-ACAP 008/2557).

Parboiled germinated brown rice (PGBR), brown rice (BR), and white rice (WR) were produced from a rice variety grown in Thailand, Khao Dawk Mali 105 *(Oryza sativa* L. ssp. *indica* cv. KDML 105). PGBR was prepared according to the method developed by Rattanadee et al. (2011) [12]. In brief, all three types of rice were cooked in an electric rice cooker (1.8 L, Sharp KS-19ET) and then freeze-dried (Supplementary Table S1).

The freeze-dried rice powders were stored at −20 °C until use. The proximate compositions of the powders are presented in the Supplementary Materials. The AIN76A diet (basal diet) was composed of corn starch (15%, *w*/*w*), casein (20%, *w*/*w*), DL-methionine (0.3%, *w*/*w*), sucrose (49.9%, *w*/*w*), corn oil (5%, *w*/*w*), cellulose powder (5%, *w*/*w*), mineral mix (3.5%, *w*/*w*), vitamin mix (1%, *w*/*w*), and choline bitartrate (0.2%, *w*/*w*). The rice powders (PGBR, BR, and WR) replaced corn starch in the AIN76A diet (15%) with ad libitum feeding and became the PGBR, BR and WR diets, respectively. The components of each diet were designed based on the macronutrient compositions in the rice (Supplementary Tables S2 and S3).

### 2.2. Experimental Design

**Figure 1:**
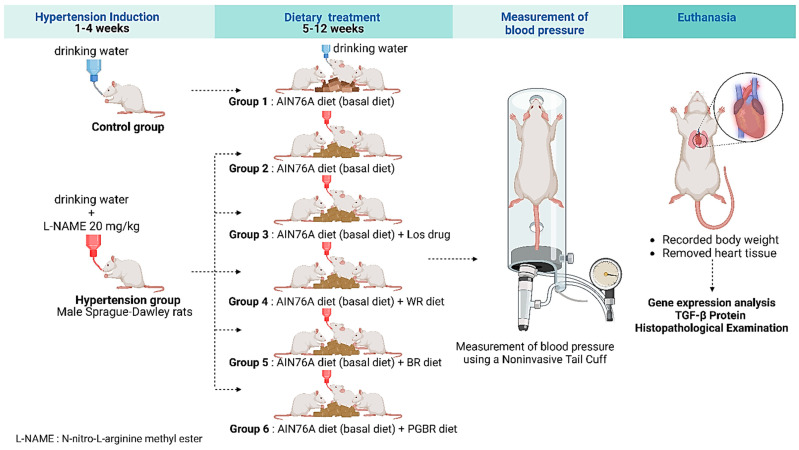
A schematic of the experimental design for inducing hypertension in rats and administering PGBR. The hypertensive groups were given *N*-nitro-L-arginine methyl ester (L-NAME) in the drinking water for a daily intake of 20 mg/kg throughout the experimental period (12 weeks).

The experimental design and treatment are illustrated in Figure 1. Following a one-week acclimatization period, the rats were randomly divided into six groups with seven animals each. The hypertensive groups were given *N*-nitro-L-arginine methyl ester (L-NAME) in the drinking water for a daily intake of 20 mg/kg throughout the experimental period (12 weeks). The rats started to consume the rice diets after four weeks until the end of the experimental period. Systolic blood pressures were measured weekly by the indirect method of tail-cuff occlusion in conscious animals using a Niprem 645 pressure recorder (Cibertec, Barcelona, Spain) [13].

The rats were divided into six groups of seven animals as follows:

Group 1: normotensive rats that received the basal diet (control);

Group 2: hypertensive rats that received the basal diet;

Group 3: hypertensive rats that received the basal diet and losartan (an anti-hypertensive drug);

Group 4: hypertensive rats that received the WR diet;

Group 5: hypertensive rats that received the BR diet;

Group 6: hypertensive rats that received the PGBR diet.

At the end of the treatment period of eight weeks, the animals were anesthetized by the inhalation of CO_2_. The body weight was recorded, and the heart was removed intact and weighed for each rat. The heart tissues were frozen and stored at −80 °C until analysis.

### 2.3. Gene Expression Analysis

The tissue for RNA isolation was the left ventricle, and RNA was extracted using an RNA isolation kit (Roche Diagnostics Corp., Indianapolis, IN, USA). The purity and quantity of the RNA were determined using a NanoDrop UV Spectrophotometer (Thermo Scientific, Wilmington, DE, USA). To measure the mRNA level, complementary DNA was amplified by a quantitative real-time polymerase chain reaction (Lightcycler 96) (Roche Diagnostics Corp., Indianapolis, IN, USA) using a SYBR Green kit (Roche Diagnostics Corp., Indianapolis, IN, USA). The forward and reverse primers used in this study [13,14,15,16] are shown in Supplementary Table S4. The quantitative fold changes in mRNA expression were determined relative to beta-actin (β-actin) using the 2^−ΔΔCT^ method [17].

### 2.4. Histopathological Examination

The tissue for the histology assay was the left atrium. The tissue was fixed with a 10% formaldehyde buffer and embedded in paraffin. Samples of 5 µm thickness were stained with hematoxylin and eosin, followed by examination under a light microscope (Olympus, Tokyo, Japan). A cross section of the heart was evaluated from photographs of whole tissue sections taken at 40× magnification by a pathologist at the National Laboratory Animal Center, Mahidol University, who was blinded to the treatments.

### 2.5. Statistical Analysis

Data are presented as means ± standards error of the means (SEM). Statistical comparisons between the groups were performed using the Mann–Whitney U test. All statistical analyses were performed using the Statistical Package for the Social Sciences for Windows (SPSS) version 18.0. The significance level was 0.05 (α = 0.05).

## 3. Results

### 3.1. Effects of White Rice, Brown Rice, and Parboiled Germinated Brown Rice Diets on Heart and Body Weight

At the end of the experiment, the heart and body weights of all rats were determined. Table 1 shows that the L-NAME-exposed group (hypertensive rats) acquired considerably more body weight than the control group (388 ± 7.64 vs. 316 ± 53.8 g; *p*-value < 0.05). This observation was also found in the white rice (WR), brown rice (BR), and parboiled germinated brown rice (PGBR)-treated groups. The L-NAME-treated rats gained body weight, but heart weight and heart/body weight ratio remained unaffected (Table 1). The diet consumption record of all groups is shown in Supplementary Figure S1. The rice consumption amounts in the WR, BR, and PGBR groups were 10.5 ± 1.8, 10.5 ± 2.1, and 11.0 ± 2.3 g/kg body weight, respectively.

### 3.2. Effects of White Rice, Brown Rice, and Parboiled Germinated Brown Rice Diets on Systolic Blood Pressure

**Figure 2:**
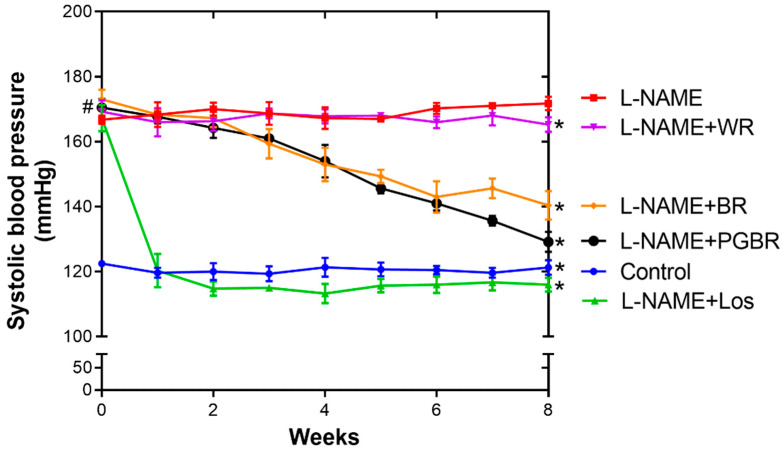
Systolic blood pressure (SBP) of rats during *N*-nitro-L-arginine methyl ester (L-NAME)-induced hypertension after hypertensive inducing for four weeks. Data are presented as means ± SEM (*n* = 7). # shows significantly different SBP in rats under different treatments compared to the control group (no treatment) at *p* < 0.05 using the Mann–Whitney U test (two-tailed), while * shows significantly different SBP in rats under different treatments compared to those treated with L-NAME (L-NAME group) at *p* < 0.05 using the Mann–Whitney U test (two-tailed). Los: losartan; WR: white rice; BR: brown rice; PGBR: parboiled germinated brown rice.

To observe the anti-hypertensive properties of the WR, BR, and PGBR treatments, rats were administered L-NAME for four weeks to assure the successful elevation of the systolic blood pressure (SBP). At week 0 in Figure 2, L-NAME significantly induced SBP in rats compared to the control group. After the successful elevation of systolic blood pressure (SBP), rats were exposed to losartan (Los, an anti-hypertensive drug), WR, BR, and PGBR for an additional eight weeks, and the SBP was measured weekly using a noninvasive tail cuff. After treatment with Los, the SBP dropped to normal within two weeks and remained constant until the end of the experiment, confirming its potent anti-hypertensive properties. Interestingly, BR- and PGBR-exposed rats showed a gradual decrease in SBP over the experimental period. PGBR was more potent than BR at week 8 (Figure 2), while the WR diet showed the smallest anti-hypertensive properties when compared with the other rice. Both the BR and PGBR diets showed anti-hypertensive properties, with PGBR being the most potent among the three types of rice.

### 3.3. Effects of White Rice, Brown Rice, and Parboiled Germinated Brown Rice on Gene Expression in Heart Tissue

**Figure 3:**
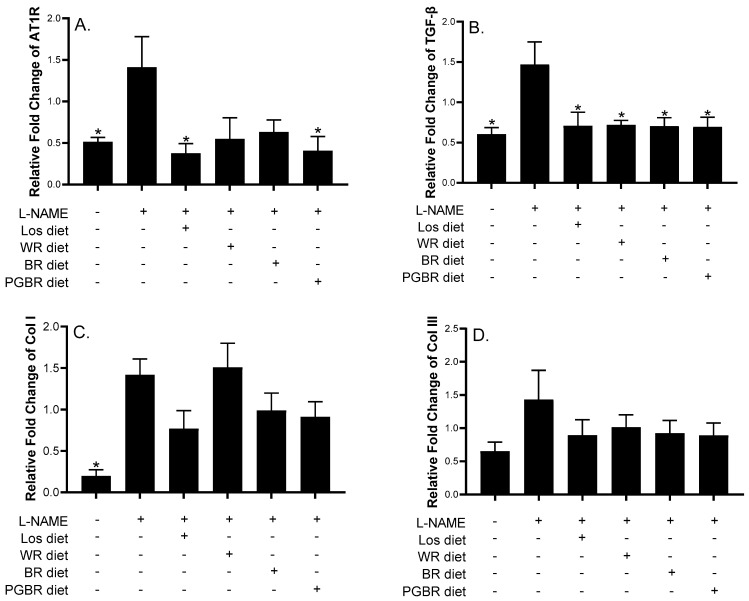
Effects of different diets on gene expression in heart tissue of rats. Data are presented as means ± SEM (*n* = 7); Gene expression of (**A**) AT1R; (**B**) TGF-β; (**C**) Col I; (**D**) Col III among rat groups. * shows significantly different relative gene expressions of rats treated with different diets compared with those treated with *N*-nitro-L-arginine methyl ester (L-NAME) at *p* < 0.05 using the Mann-Whitney U test (two-tailed). AT1R: angiotensin II type 1 receptor; TGF-β: transforming growth factor-β; Col I: collagen type I; Col III: collagen type III; Los: losartan; WR: white rice; BR: brown rice; PGBR: parboiled germinated brown rice.

**Figure 4:**
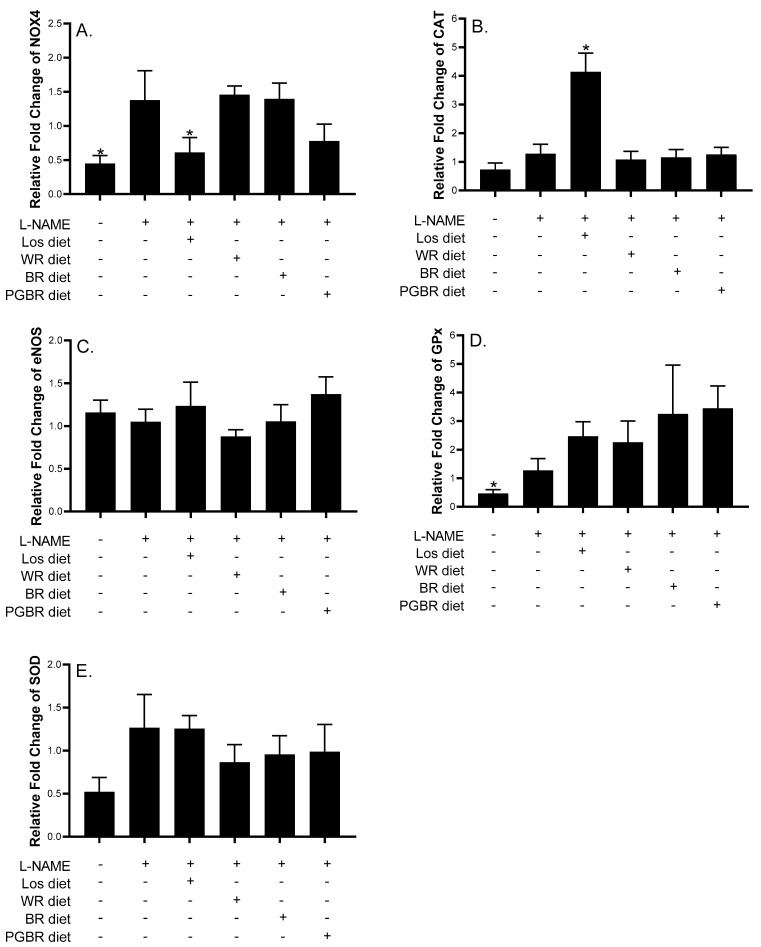
Effects of different diets on gene expression in heart tissue of rats. Data are presented as means ± SEM (*n* = 7); Gene expression of (**A**) NOX 4; (**B**) CAT; (**C**) eNOS; (**D**) GPx; (**E**) SOD among rat groups. * shows significantly different relative gene expressions of rats treated with different diets compared with those treated with *N*-nitro-L-arginine methyl ester (L-NAME) at *p* < 0.05 using the Mann-Whitney U test (two-tailed). NOX4: NADPH oxidase; CAT: catalase; eNOS: endothelial nitric oxide synthase; GPx: glutathione peroxidase; SOD: superoxide dismutase; Los: losartan; WR: white rice; BR: brown rice; PGBR: parboiled germinated brown rice.

Figure 2 shows the promising anti-hypertensive effects of BR and PGBR. Therefore, the heart tissues of PGBR-treated rats were further investigated for gene expression, including (i) the renin–angiotensin system, such as AT1R; (ii) the TGF-β, collagen type I (Col I), and collagen type III (Col III) genes, which are involved in fibrosis; (iii) the NADPH oxidase (NOX4) gene, which is involved in oxidative stress production; and (iv) antioxidant enzymes and nitric oxide-producing enzymes (glutathione peroxidase (GPx), catalase (CAT), superoxide dismutase (SOD), and endothelial nitric oxide synthase (eNOS)). Figure 3 and Figure 4 demonstrate that L-NAME-treated rats showed significantly increased gene expression of AT1R, TGF-β, Col I, and GPx. In the Los-exposed group, the expression of AT1R, TGF-β, and NOX4 decreased, even though the expression of most antioxidant enzymes remained unchanged, indicating that in our condition AT1R, TGF-β, and NOX4 might be therapeutic targets for Los in the treatment of hypertension. Interestingly, the PGBR-treated group significantly suppressed AT1R and TGF-β expression at the same level as the control and Los-treated groups (Figure 3). Although NOX4 was not statistically reduced, a clear trend of reduction was obtained. Therefore, PGBR might lower SBP by targeting AT1R, TGF-β, and NOX4, similar to the anti-hypertensive drug (Los), while the antioxidant enzymes may be neglected.

### 3.4. Effect of PGBR on Heart Histopathological Changes

**Figure 5:**
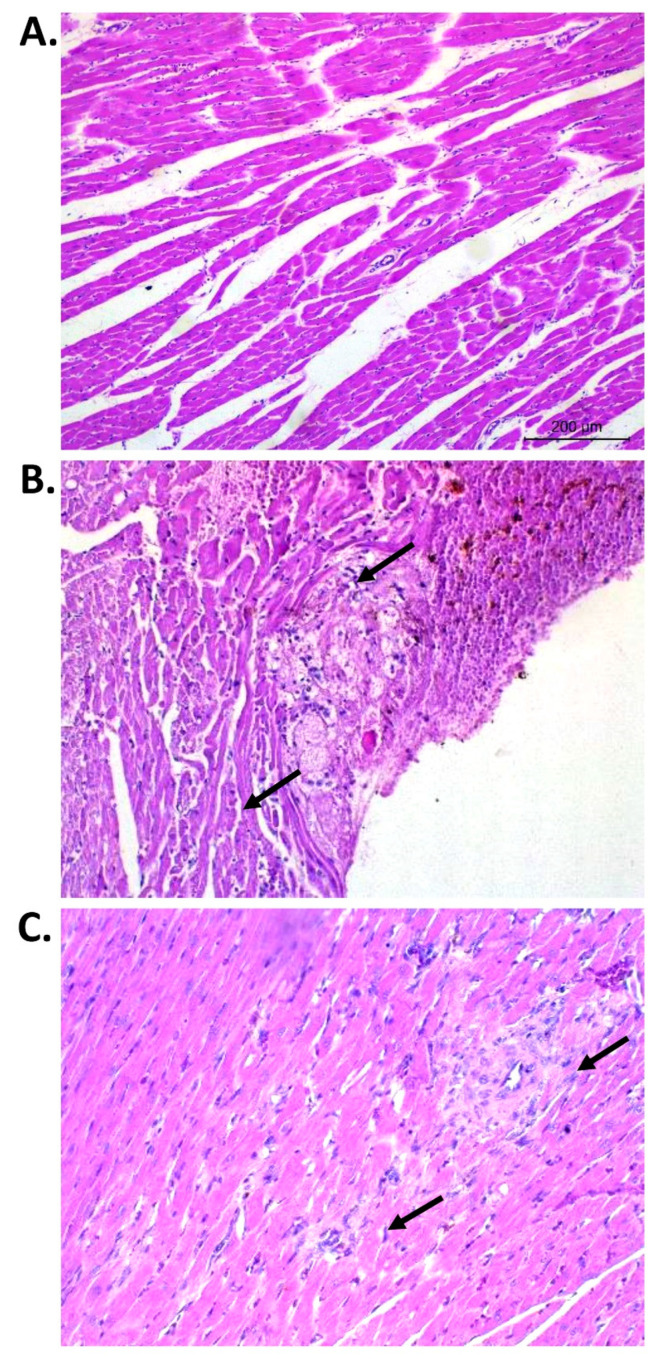
Histopathological changes in the heart tissues. H&E staining under a light microscope: (**A**) a representative figure of normal myocardial fibers: 10×, (**B**) a representative figure of focal myocardial necrosis and inflammatory infiltration: 40×, and (**C**) a representative figure of myocardial fibrosis and scar formation: 40×. The arrows indicate cardiomyopathy in the heart tissue.

The heart wall is composed of three layers, from the inside to the outside: the endocardium, myocardium, and epicardium. Several types of lesions are induced during hypertension. Thus, to ensure the anti-hypertensive properties of PGBR, we performed a histopathological analysis using H&E staining for lesion quantification. Representative figures of normal myocardial fibers, myocardial necrosis, inflammatory infiltration, and myocardial fibrosis and scar formation, which were scored in the present study, are shown in Table 2 and Figure 5. In the L-NAME group, lesions were found in all layers, with focally endothelial hyperplasia in the epicardium. In the myocardium, cellular degeneration to necrosis in inflammatory cells was observed. In the sub-endocardium, large focal branching fibrosis, acute to chronic hemorrhages, and focal collagen deposits were frequently found. The outer epicardium showed cellular hyperplasia with mimic mononuclear cell infiltration. A cardiomyopathy analysis revealed degeneration, necrosis, inflammatory cell infiltration, and fibrosis/collagen scar formation. Treatment with PGBR in hypertensive rats showed a lower amount of cardiomyopathy compared with the L-NAME-induced hypertensive rat group, indicating an improvement in cardiac histology from PGBR after hypertension.

## 4. Discussion

This study reports the anti-hypertensive effect of parboiled germinated brown rice (PGBR) made from the Khao Dawk Mali 105 variety on antioxidant enzymes and gene expression associated with fibrosis in the hearts of hypertensive rats induced by *N*-nitro-L-arginine-methyl ester (L-NAME). Clear beneficial results were obtained when the diet was mixed with PGBR. The treatment with PGBR attenuated blood pressure and decreased myocardial degeneration. In several studies, there were no effects of L-NAME on body weight [18,19]. However, increased body weight was observed in the L-NAME group in this study. It could be that the three rice groups of rats consumed higher amounts of food than the control, L-NAME, and drug groups. Therefore, the weights were higher than in the other groups (Supplementary Figure S1). Moreover, the three different rice groups showed increases in body weight. These might be the effects of the difference in energy from the rice diets. The corn starch in the basal diet was substituted with different rice varieties. Therefore, the difference in energy in the rat diets came from the rice types. The rats in the WR, BR, and PGBR groups received 40.0 ± 6.9, 40.8 ± 8.1, and 42.8 ± 9.1 kcal of energy from rice, respectively (Supplementary Table S2).

Previous studies reported that PGBR contained high amounts of γ-aminobutyric acid (GABA), ferulic acid, γ-oryzanol, γ-tocotrienol, and p-coumaric acid and showed higher antioxidant abilities than white rice and brown rice [9,10]. In agreement with earlier reports, the anti-hypertensive effect of pregerminated brown rice was also evaluated in spontaneously hypertensive rats (SHRs) fed a diet containing 40% pregerminated brown rice for eight weeks. The results showed anti-hypertensive effects compared to the control group [20]. In this study, L-NAME-induced hypertensive rats showed a significantly increased systolic blood pressure (SBP) that significantly decreased after treatment with PGBR. The results agreed with the anti-hypertensive effect of PGBR, which contains high contents of bioactive ingredients [9,20], especially GABA and ferulic acid. The mechanism of the hypotensive action of the administered GABA has not yet been fully explained, but GABA showed an anti-hypertensive action as an inhibitory neurotransmitter in the central nervous system [14], while a low dose of a novel cultivar of GABA-rich tomato had an anti-hypertensive effect in SHRs by reducing SBP compared to the control group in both single- and chronic-administration studies [21].

The administration of L-NAME, an L-arginine analog, produces hypertension, vascular resistance, hypertrophy, myocardial remodeling, and vasoconstriction. Angiotensin II (Ang II) influences the phases of the inflammatory response involved in the mechanisms leading to vascular remodeling by stimulating vascular repairs such as transforming growth factor-β1 (TGF-β1), hypertrophy, the accumulation of extracellular matrix, and collagen deposition [17]. Thus, inflammation seems to be closely related and might mediate Ang-II-induced vascular remodeling. Ang II and TGF-β1 stimulate the development of cardiac hypertrophy, myocardial fibrosis, the accumulation of collagen type I (Col I) and collagen type III (Col III), fibrosis, and the structural organization of collagen. The effects of Ang II are either indirect by the upregulation of TGF-β1 expression or direct by Ang II receptors: AT1R [6,20]. All these reports showed relationships among AT1R, TGF-β1, Col I, and Col III that may contribute to myocardial fibrosis in the animal model [22]. This study demonstrated that treatment with PGBR in L-NAME-induced hypertensive rats significantly decreased AT1R, TGF-β1, and Col I gene expression compared to the L-NAME group. The findings indicated that the anti-inflammatory effect of PGBR improved myocardial fibrosis by inhibiting genes involved in the renin–angiotensin system and fibrosis.

Vasoconstriction is also a mechanism leading to hypertension, while the chronic inhibition of nitric oxide synthase (NOS) may lead to vasoconstriction [18,19]. Nitric oxide (NO) is synthesized in endothelial cells from L-arginine and is converted to NO by endothelial nitric oxide synthase (eNOS). Moreover, the inhibition of eNOS activity leads to increased expression of nicotinamide adenine dinucleotide phosphate (NADPH) oxidase (NOX4), a major source of reactive oxygen species (ROS) in vascular tissue. The accumulation of reactive oxygen species decreased antioxidant defense systems and reduced NO bioavailability [23,24,25]. The L-NAME treatment decreased the expression of the endothelial nitric oxide synthase (eNOS) gene after long-term L-NAME treatment, while many studies reported increased eNOS mRNA levels in the heart and kidney in hypertensive rats [26]. Moreover, L-NAME induced hypertension by involving the renin–angiotensin aldosterone system (RAAS) [27]. It was observed that an L-NAME treatment raised eNOS protein levels [26]. However, in our work, L-NAME had no effect on eNOS mRNA, which might indicate that mRNA is dependable for early responses. Further investigation into the eNOS protein level may be worth pursuing. In this study, treatment with PGBR tended to increase eNOS expression and progressively decreased NOX4 gene expression in the hearts of L-NAME-treated rats, but no significant differences were observed. Therefore, the mechanisms of PGBR that contain bioactive ingredients may be involved in the antioxidant activity and preserve the bioavailability of NO in hypertensive hearts.

The relationship between the development of hypertension, decreased antioxidant capacity, and the increased bioavailability of ROS has been indicated in many experimental models [28]. Major enzymatic antioxidant defenses include superoxide dismutase (SOD), glutathione peroxidase (GPx), and catalase (CAT) as the primary lines of cellular defense against oxidative damage [29]. In this study, only GPX expression was induced after L-NAME treatment, while CAT and SOD expression were not different between the control and L-NAME groups, suggesting that GPx might be the main enzyme quenching oxidative stress in hypertensive rats. This effect displayed a compensatory mechanism to attenuate the excessive ROS production [30]. This result agreed with Kumar et al. (2012) [31], who found improved GPx activity after treatment with syringic acid in L-NAME-induced hypertensive rats. The increased activity of enzymatic antioxidants might be due to free radical scavenging efficacy as a beneficial action against pathophysiological modifications caused by superoxide anions and hydroxyl radicals [21]. These antioxidant genes appeared to fluctuate without being statistically significant. For example, a recent study determined that lead-induced hypertension had no effect on SOD, CAT, or GPx in the hearts of the animals but increase NOX4 [32], and another study showed that SOD, CAT, and GPx were not changed in the kidneys of hypertensive rats (measured at week 16) [33].

As the L-NAME raised the expression of NOX4 in L-NAME-treated rats, resulting in an increase in oxidants, we hypothesized that alternative antioxidant enzymes may play an important role in mitigating the oxidative stress caused by L-NAME.

The inflammatory response and cytokines are important components of the host response to heart injury from hypertension and play a key role in cardiac repair [34], ultimately leading to the replacement of dead myocardium with a collagen-based scar and distorted architecture and function of the heart. Moreover, excess collagen deposition and fibrosis have been linked to myocardial stiffness and systolic and diastolic abnormalities [35,36], suggesting that interstitial myocardial fibrosis may be related to L-NAME-induced vasoconstriction with consequent myocardial ischemia. In this study, hematoxylin and eosin (H&E) staining was used for the test to examine rat tissue for cardiomyopathy. Some studies reported that the histology results related to biochemical parameters and ECG results [37,38,39]. In this study, rats could be induced to be hypertensive, promoting myocardium inflammation and scar deposition. However, when comparing the microscopic findings (Table 2), collagen involving scars, fibrosis in the myocardium, and collagen deposit in the sub-endocardium were not observed in the three rice groups. Only one lesion was found in the L-NAME group. It is possible that the rats had no severity in their heart pathologies, so the markers, such as gene expression, did not show any differences among the rice groups. Our data showed that the PGBR group seemed to have the lowest downregulation in scar deposition gene expression, such Col I and III. Thus, the administration of PGBR not only inhibited the renin–angiotensin system and fibrosis but also improved cardiac histology.

The results from this study suggested that the ingestion of PGBR might protect the heart against L-NAME-induced hypertension. The molecular mechanism of how PRBR reduced hypertension might be (i) the inhibition of the renin–angiotensin axis, (ii) the inhibition of fibrosis, or (iii) the inhibition of oxidative-stress-generating enzymes (NOX) and the activation of an antioxidant enzyme (GPx). Thus, the consumption of PGBR might reduce heart damage from hypertension pathology in this hypertensive rat model. Some study limitations were observed. The diastolic blood pressure and mean arterial pressure were not recorded, while the protein expression for several genes was not measured. The variations in oxidative stress and antioxidant genes were observed with no statistical findings. A limitation in this study could be that the food was freely accessed by the rats. The exact rice doses were difficult to calculate. Further studies should investigate more biomarkers, such as antioxidant or anti-inflammatory markers, to further clarify the mechanism of PGBR for the treatment of hypertension.

## 5. Conclusions

This is the first report demonstrating the anti-hypertensive properties of parboiled germinated brown rice (PGBR) in *N*-nitro-l-arginine methyl ester (L-NAME)-induced hypertensive rats. PGBR significantly decreased the angiotensin II type 1 receptor and transforming growth factor-β and significantly increased glutathione peroxidase expression. The gene expression of nicotinamide adenine dinucleotide phosphate oxidase 4 and collagen type I progressively decreased in the PGBR group. Thus, the regular consumption of PGBR could treat hypertension as in this animal model study.

## Figures and Tables

**Table 1:** Heart and body weights of rats after different treatments.

Group	Body Weight (g)	Heart Weight (g) ^NS^	Heart/Body Weight ratio (g %) ^NS^
Control	315 ± 53.8 *	1.06 ± 0.06	0.34 ± 0.02
L-NAME	388 ± 20.2	1.10 ± 0.07	0.28 ± 0.02
L-NAME + Los	401± 10.0	1.39 ± 0.11	0.35 ± 0.03
L-NAME + WR	408 ± 9.00 *	1.29 ± 0.08	0.32 ± 0.02
L-NAME + BR	413 ± 21.3 *	1.17 ± 0.07	0.28 ± 0.01
L-NAME + PGBR	422 ± 27.3 *	1.23 ± 0.07	0.29 ± 0.02

All data are presented as means ± SEM (*n* = 7). The * shows significantly different weights of rats under different treatments at *p* < 0.05 compared to those treated with *N*-nitro-L-arginine methyl ester (L-NAME) using the Mann–Whitney U test (two-tailed). ^NS^ shows no significant difference among rat groups. Los: losartan; WR: white rice; BR: brown rice; PGBR: parboiled germinated brown rice.

**Table 2:** Microscopic findings of animals in each group, with numbers of heart lesions.

Microscopic Findings	Control	L-NAME	L-NAME + Los	L-NAME + WR	L-NAME + BR	L-NAME + PGBR
Fibrosis, myocardium	0	1	0	0	0	0
Degeneration to necrosis, myocardium	1	1	0	0	1	0
Inflammation to Aschoff body cell infiltration	0	1	2	1	0	0
Collagen deposit, sub-endocardium	0	1	0	0	0	0
* Cardiomyopathy	1	3	2	1	1	0

* Cardiomyopathy combines degeneration, necrosis, inflammatory cell infiltration, and fibrosis/collagen scar formation; a refers to the same animal, number 2/1 *(n =* 5). L-NAME: *N*-nitro-L-arginine methyl ester; Los: losartan; WR: white rice; BR: brown rice; PGBR: parboiled germinated brown rice.

## Data Availability

All data are contained within this article and the Supplementary Materials.

## Supplementary Materials

The following supporting information can be downloaded at: https://www.mdpi.com/article/10.3390/foods12010009/s1, Supplementary Table S1: The condition for rice cooking; Supplementary Table S2: The compositions of cooked rice powders; Supplementary Table S3: The composition of the basal formula diet (AIN-76A); Supplementary Table S4: Primers used for amplification in real-time PCR; Supplementary Figure S1: Diet consumption in all animal groups.
