# Supplementary material for: Parboiled Germinated Brown Rice Improves Cardiac Structure and Gene Expression in Hypertensive Rats
Source: Foods. 2022 Dec 20;12(1):9. doi: 10.3390/foods12010009 (PMC9818593; doi:10.3390/foods12010009)

foods

Supplementary Materials

Parboiled Germinated Brown Rice Improves Cardiac Structure and Gene Expression in Hypertensive Rats

Nattira On-Nom [1], Kanoknad Khaengamkham [1], Aikkarach Kettawan [1], Thanaporn Rungruang [2], Uthaiwan Suttisansanee [1], Piya Temviriyanukul [1], Pattaneeya Prangthip [3] and Chaowanee Chupeerach [1,*]

[1] Institute of Nutrition, Mahidol University, Salaya, Phuttamonthon, Nakhon Pathom 73170, Thailand
[2] Department of Anatomy, Faculty of Medicine Siriraj Hospital, Mahidol University, Bangkok 10700, Thailand
[3] Department of Tropical Nutrition and Food Science, Faculty of Tropical Medicine, Mahidol University, Ratchathewi, Bangkok 10400, Thailand
* Correspondence: chaowanee.chu@mahidol.ac.th

Supplementary Table S1: The condition for rice cooking

Three hundred grams of WR, BR, and PGBR were cooked using an electronic rice cooker, Sharp KS-19ET model, size 1.8 liters (Sharp, Japan). Each steamed rice was cooked under difference conditions. Cooked rice samples were analyzed nutrients and energy (supplementary Table S1). Then, the rice samples were freeze-dried and grinded into rice flour. After that, PGBR, BR, and WR diets were prepared by replacing 15% corn starch in the basal formula diet (AIN 76A) (supplementary Table S2). with freeze-dried cooked PGBR, BR, and WR powders, respectively. The content of each rice added into the diet were based on the macronutrient compositions and energy as displayed in Table S3.

Rice	Condition for cooking
Parboiled germinated brown rice (PGBR)	PGBR was cooked with a 1.0 : 2.3 rice to water ratio, at 100 °C, simmered for 33 min, and set to well-cooked for 15 min.
Brown rice (BR)	BR was cooked with a 1.0 : 2.2 rice to water ratio, at 100 °C, simmered for 30 min, and set to well-cooked for 15 min.
White rice (WR)	WR was cooked with a 1.0 : 1.8 rice to water ratio, at 100 °C, simmered for 15 min, and set to well-cooked for 15 min.

Supplementary Table S2: The compositions of cooked rice powders

Major composition/100 g	WR	BR	PGBR
Energy (Kcal)	382.6	389.5	389.2
Protein (g)	6.6	7.1	6.9
Fat (g)	0.4	2.4	2.6
Carbohydrate (g)	88.2	84.9	84.4
γ-amino butyric acid (GABA) (mg)	1.2	2.6	11.9
γ-oryzanol (mg)	ND	11.4	13.3
Total phenolic acid (mg)	8.4	37.1	69.6
Total vitamin E (mg)	ND	1.17	1.19
Dietary fiber (g)	0.8	3.0	3.5

*ND = not detected

Supplementary Table S3: The compositions of basal formula diet (AIN-76A)

Diet ingredients	Percent
Sucrose	50
Casein-vitamin free	20
Corn starch	15
Powdered cellulose	5
Corn oil	5
AIN-76 mineral mix	3
AIN-76 vitamin mix	1
DL-methionine	0.3
Choline bitartrate	0.2

Supplementary Table S4: Primers used for amplification by Real-Time PCR

Gene	Forward primer (5′-3′)	Reverse primer (5′-3′)	Annealing temp. (°C)	Amplified length (bp)
eNOS	GGGCCAGGGTGATGAGCTCTG	CCCTCCTGGCTTCCAGTGTCC	64	323
NOX4	TTGCTTTTGTATCTTC	CTTACCTTCGTCACAG	64	243
AT1R	CACCTATGTAAGATCGCTTC	GCACAATCGCCATAATTATCC	64	211
TGF-β	CCGCAACAACGCAATCTATG	AGCCCTGTATTCCGTCTCCTT	64	304
Col I	TCAGGGGCGAAGGCAACAGT	TTGGGATGGAGGGAGTTTACACGA	64	218
Col III	CGTCCTGCAGGTAACAGTGGTTC	TGCTCCAGTTAGCCCTGCAA	64	148
SOD	CGTCATTCACTTCGAGCAGAAGG	GTCTGAGACTCAGACCACATA	64	443
CAT	ACAACTCCCAGAAGCCTAAGAATG	GCTTTTCCCTTGGCAGCTATG	58	76
GPx	GGAGAATGGCAAGAATGAAGA	CCGCAGGAAGGTAAAGAG	60	138
β-actin	TGGCTCATCGTAGGGAGTTT	CTCGTCCTCGACTGAGAAGC	64	121

Supplementary Figure S1: Diet consumption in all animal groups

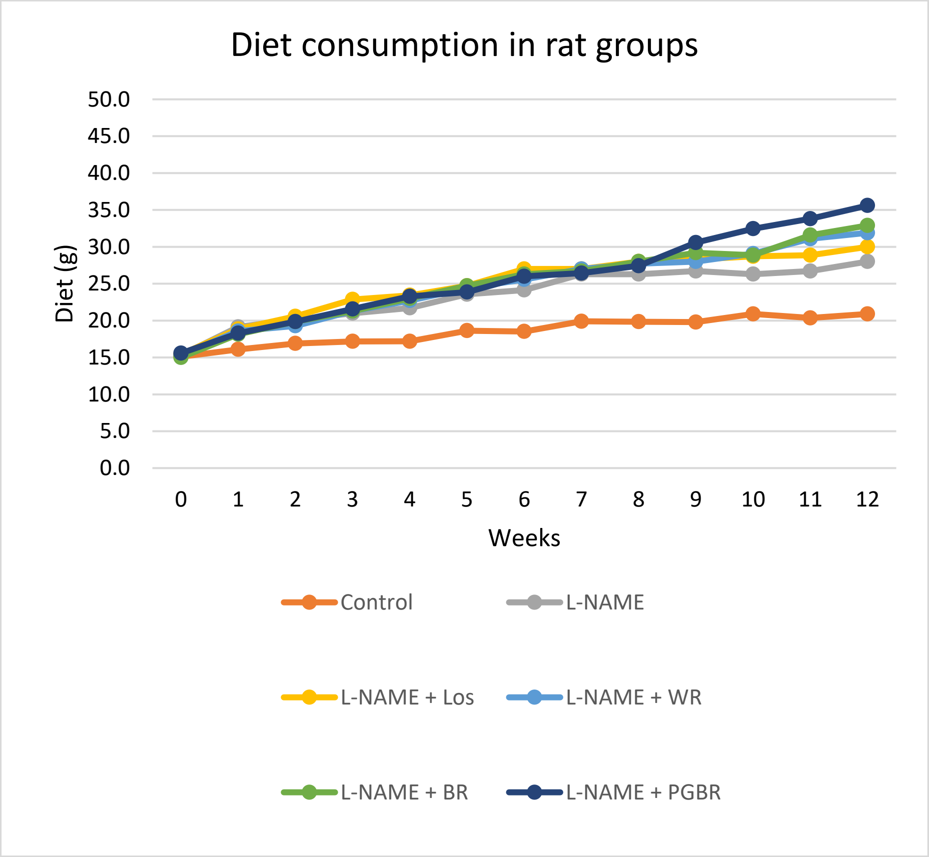

Supplement: Supplementary file 1 [file foods-12-00009-s001.zip › foods-1973906-supplementary.pdf]
